# Supplementary material for: Development of a curricular thread to foster medical students’ critical reflection and promote action on climate change, health, and equity
Source: PLoS One. 2024 May 30;19(5):e0303615. doi: 10.1371/journal.pone.0303615 (PMC11139332; doi:10.1371/journal.pone.0303615)
Supplement: Attachment — Submitted filename: Response to Reviewers.docx [file pone.0303615.s001.docx]

March 31, 2024

*PLOS ONE*

Dear Dr. Chenette,

We are pleased to submit our revised manuscript now entitled, “Development of a curricular thread to foster medical students’ critical reflection and promote action on climate change, health, and equity.”

We are greatly appreciative of the comments we received from the three reviewers. As we hope you will see, by incorporating suggestions, we have significantly improved our manuscript.

Our manuscript focuses on a timely curricular innovation undertaken at our medical school – the incorporation of climate change, health, and equity topics into undergraduate medical education. We describe the needs assessment; development of a community of practice; and design and execution of curricular materials, including lectures, discussions, experiential learning opportunities in the community, and a critical reflection essay. A total of 118 first-year medical students participated in the thread and we thoroughly analyzed the critical reflection essays for themes and by the REFLECT rubric.

While revising this manuscript, we have now included substantial details in our methods and results sections, included suggested citations by the reviewers, and revised the flow of the writing. Most notably, we have 1) added one figure summarizing the results of the critical reflection essays according to the REFLECT rubric and 2) included four tables. The first table outlines all our curricular activities in detail, as requested by the reviewers, including the lecture title, lecturer name and affiliation, learning objectives, and Bloom’s Taxonomy domains addressed. The remaining three tables conveys the themes found in the critical reflection essays according to Bloom’s Taxonomy and the socio-ecological model. We have included numerous direct quotes to convey the themes and highlight the efficacy that students felt this thread had on their learning, reflection, and growth. We hope that by including all new materials, readers may consider implementing this thread at their own institutions, which is a necessity given the pressing nature of climate change and its health impacts.

Our findings highlight that medical students synthesize information about climate change into their medical knowledge and can reflect on the affective and actionable elements of climate change in relation to human health. We wish to underscore not only the enthusiasm of students who participated but also the student-driven nature of the thread development itself and writing of this manuscript. Altogether, our curricular thread depicts the fruitful roles the next generation of physicians are taking in further medical education innovations.

The total word count of our abstract is 296 words. Our manuscript is approximately 3,400 words (excluding abstract, references, tables, figure, and appendix). All authors on this manuscript have contributed substantially to the work including conceptualizing the purpose, reviewing the literature, and writing the manuscript. None have conflicts of interest to declare.

Sincerely,

Trisha Dalapati

Medical Student

Duke University School of Medicine

| Comments | Response | Page # |
| --- | --- | --- |
| 1. Please ensure that your manuscript meets PLOS ONE's style requirements, including those for file naming. | We acknowledge that we have reviewed the PLOS ONE style requirements. |  |
| 2. Note from Emily Chenette, Editor in Chief of PLOS ONE, and Iain Hrynaszkiewicz, Director of Open Research Solutions at PLOS: Did you know that depositing data in a repository is associated with up to a 25% citation advantage ([https://doi.org/10.1371/journal.pone.0230416)?](https://urldefense.com/v3/__https:/doi.org/10.1371/journal.pone.0230416)?__;!!OToaGQ!pw9o1zIuzQqFSDFqTKBOr4O6KL0sIdf5bKWu4D8G9LfsURriVV4h90BACFu_m93ulEBYs2qX4rvDFjlbIB0nug$) If you’ve not already done so, consider depositing your raw data in a repository to ensure your work is read, appreciated and cited by the largest possible audience. You’ll also earn an Accessible Data icon on your published paper if you deposit your data in any participating repository ([https://plos.org/open-science/open-data/#accessible-data](https://urldefense.com/v3/__https:/plos.org/open-science/open-data/*accessible-data__;Iw!!OToaGQ!pw9o1zIuzQqFSDFqTKBOr4O6KL0sIdf5bKWu4D8G9LfsURriVV4h90BACFu_m93ulEBYs2qX4rvDFjnGGoqzkA$)). | We thank the editor for this comment and agree wholeheartedly with the importance of making raw data accessible. It was not within our IRB to publish the full essays of the student-participants in this thread. Therefore, to be as transparent as possible, we have added three tables (Table 2-4) that shows numerous direct quotes from student essays, which support our findings and conclusions. | Pages 22-27 |
| 3. Please provide additional details regarding participant consent. In the ethics statement in the Methods and online submission information, please ensure that you have specified (1) whether consent was informed and (2) what type you obtained (for instance, written or verbal, and if verbal, how it was documented and witnessed). If your study included minors, state whether you obtained consent from parents or guardians. If the need for consent was waived by the ethics committee, please include this information.  Once you have amended this/these statement(s) in the Methods section of the manuscript, please add the same text to the “Ethics Statement” field of the submission form (via “Edit Submission”). | We appreciate this comment by the reviewers. We have added more detail regarding the participant consent in the Methods section. It reads as follows:  “The Duke University Institutional Review Board determined data collected for the Climate Change, Health, and Equity curricular thread was exempted educational research. All students participating in the curricular thread were informed of the educational research. Students were required to complete the critical reflection essay for the FPC2 course. However, they could opt out of having their essay included in this analysis by contacting an assigned individual at DUSOM, who was not involved with the thread; if the students did not contact the assigned individual, consent to have their essay analyzed was assumed. We adhered to the Standards for Reporting Qualitative Research.” | Lines 5-12, Page 10 |
| 4. We note that your Data Availability Statement is currently as follows: [All relevant data are within the manuscript and its Supporting Information files.]  Please confirm at this time whether or not your submission contains all raw data required to replicate the results of your study. Authors must share the “minimal data set” for their submission. PLOS defines the minimal data set to consist of the data required to replicate all study findings reported in the article, as well as related metadata and methods ([https://journals.plos.org/plosone/s/data-availability#loc-minimal-data-set-definition](https://urldefense.com/v3/__https:/journals.plos.org/plosone/s/data-availability*loc-minimal-data-set-definition__;Iw!!OToaGQ!pw9o1zIuzQqFSDFqTKBOr4O6KL0sIdf5bKWu4D8G9LfsURriVV4h90BACFu_m93ulEBYs2qX4rvDFjk03k3wpQ$)). | We request to revise this statement to “All relevant data are within the manuscript.” We have included our qualitative data in Tables 2-4. It was not within our IRB to publish the full essays of the student-participants in this thread. These three tables (Table 2-4) that shows numerous direct quotes from student essays, which support our findings and conclusions. | Pages 22-27 |
| 5. Please ensure that you include a title page within your main document. You should list all authors and all affiliations as per our author instructions and clearly indicate the corresponding author. | We acknowledge that we have done this. | Page 1 |
| 6. Please amend your authorship list in your manuscript file to include authors Andrew Muzyk, Trisha Dalapati, Sneha Mantri, Phillip Mitchell, Ian Allen George, Kathryn Andolsek, John Matthew Velkey, and Jennifer Lawson. | We acknowledge that we revised our authorship list and included the title page within the manuscript file. | Page 1 |
| 7. Please amend either the abstract on the online submission form (via Edit Submission) or the abstract in the manuscript so that they are identical. | We acknowledge that we revised our abstract and included the revised version within the manuscript file. | Page 2-3 |
| 8. Please include your full ethics statement in the ‘Methods’ section of your manuscript file. In your statement, please include the full name of the IRB or ethics committee who approved or waived your study, as well as whether or not you obtained informed written or verbal consent. If consent was waived for your study, please include this information in your statement as well. | We have included the following in our manuscript Methods section:  “The Duke University Institutional Review Board determined data collected for the Climate Change, Health, and Equity curricular thread was exempted educational research. All students participating in the curricular thread were informed of the educational research. Students were required to complete the critical reflection essay for the FPC2 course. However, they could opt out of having their essay included in this analysis by contacting an assigned individual at DUSOM, who was not involved with the thread; if the students did not contact the assigned individual, consent to have their essay analyzed was assumed. We adhered to the Standards for Reporting Qualitative Research.” | Lines 5-12, Page 10 |
| 1. Is the manuscript technically sound, and do the data support the conclusions?  The manuscript must describe a technically sound piece of scientific research with data that supports the conclusions. Experiments must have been conducted rigorously, with appropriate controls, replication, and sample sizes. The conclusions must be drawn appropriately based on the data presented.  Reviewer #1: Partly  Reviewer #2: Yes  Reviewer #3: Partly | We appreciate the reviewers’ comments. We acknowledge that in several areas of our results we could have included more direct quotes to support our findings and conclusions. We also wholeheartedly agree with the reviewer’s comments that we should have included more details on the curricular activities, including lectures, to facilitate reproducing this type of curricular thread at another institution. To address these important concerns, we have made a table to outline the thread activities (Table 1) and tables of direct quotes from student essays to underscore the thematic analysis conclusions. | Page 19-27 |
| 2. Has the statistical analysis been performed appropriately and rigorously?  Reviewer #1: I Don't Know  Reviewer #2: N/A  Reviewer #3: No | We appreciate the reviewer’s comments. We agree with reviewer 2 that this comment is not largely applicable to our research study which is qualitative in nature. |  |
| 3. Have the authors made all data underlying the findings in their manuscript fully available? Reviewer #1: Yes  Reviewer #2: Yes  Reviewer #3: No | We recognize that Reviewer 3 did not feel that we adequately made all underlying findings available. As previously mentioned, we have now included our qualitative data in Tables 2-4. It was not within our IRB to publish the full essays of the student-participants in this thread. We hope that addition of these three tables (Table 2-4) showing direct quotes from student essays will address reviewer 3’s concerns. | Page 22-27 |
| Reviewer #1:  Overall, this paper has utility for educators at other institutions interested in creating more climate content for their students. It is unclear how much of this one example is applicable generally, since a unique feature of the effort at this institution is that the existing course directors played a key part in introducing new content. More importantly, the information about the course may be too scant to be useful. And not enough detail warrants much more detail, which isn’t feasible in this sort of paper. I would thus streamline the paper to the crucial, transferable content, eliminating details that are not relevant to the overall architecture of the course, which will also make this paper more readable. I hope that my specific comments can provide some additional guidance. | We thank the reviewer for recognizing the utility of this manuscript in helping other institutions create climate content for their students. Certainly, we acknowledge the key role of the course directors in developing and presenting this content to students. We hope that this manuscript can serve as a guide to creating a similar climate thread at other institutions, lowering the barrier to institute new content, and making it easier for course directors and other institutional leaders to present similar thread content to students.  For other institutions, we agree that detail is critical for reproducibility and even improvement upon the thread that we have created. For this reason, we have edited the manuscript throughout to emphasize the transferable content. Additionally, we have added more detail to Table 1 (“Overview of curricular thread activities”), which now includes lecturer affiliations, session lengths, as well as objectives, themes, and Bloom’s Taxonomy domains addressed by each thread component. We have addressed specific comments below. | Page 19-21 |
| Reviewer #1:  Of note, the writing could use the librarian’s once-over. “At present” not “presently”. “Effect” not “affect”; “synthesize: not “synthesize ..together” etc. And that’s just the opening section | We thank the reviewer for pointing out these errors. The manuscript has been thoroughly revised for grammar and clarity. |  |
| Reviewer #1:  p. 1 “surrounding environment” This is vague and can be anything. Please be clear about what aspects of the surrounding environment you mean, as this orients your argument. | Thank you for this comment. We have specified that “surrounding environment, including the climate, geography, and healthcare inequities in the community.” | Page 4, line 4-5 |
| Reviewer #1:  Building teaching capacity… financial support” Consider including: Blanchard OA, Greenwald LM, Sheffield PE. The Climate Change Conversation: Understanding Nationwide Medical Education Efforts. Yale J Biol Med. 2023 ;96(2):171-184. | We thank the reviewer for pointing us to this important paper. We have added in Blanchard et al.’s article as reference 12. | Page 5, line 7 |
| Reviewer #1:  “Though leaders have recommended learning objectives…” Consider including: ] Sorensen C. Campbell H, Depoux A. et al. Core Competencies to Prepare Health Professionals to Respond to the Climate Crisis. Plos Climate 2023; 2(6): e0000230. | We thank the reviewer for pointing us to this important paper. We have added in Sorensen et al.’s article as reference 18. | Page 5, line 12 |
| Reviewer #1:  “Considering the education need and expert recommendation” It’s very important to be precise here, as this represents the transition from the previous general overview/state of climate education to this institution’s response. What exactly was the CoP convened to address? Start this paragraph with “In order to address…” Then state what the goal of the CoP was (e.g. In order to address the need for a more robust climate curriculum with measurable goals, we…” | We strongly agree with the reviewer’s comment that we were initially not clear nor precise with the goal of the CoP. Therefore, we have revised the paragraph to address this issue:  “In fall 2022, faculty members and trainees met to address the lack of UME at our institution, Duke University School of Medicine (DUSOM), focused on climate change and its impacts on health. Subsequently, we created a community of practice (CoP) of medical educators and students. The CoP’s purpose was to identify areas within the first-year medical curriculum where topics of climate change could be integrated and to then develop a curricular thread in the first-year medical program with clear objectives and measurable outcomes. The resulting curricular thread was named Climate Change, Health, and Equity.” | Page 5, line 13-19 |
| Reviewer #1:  How were the learning objectives and goals derived (based on what sources?) | In Table 1 (“Overview of Curricular Thread Activities”), we outline the curricular objectives and themes from each activity that was planned for the curricular thread. These learning objectives and goals were based on each lecturer’s expertise and were reviewed by the larger CoP. | Page 19-21, Column labeled “Objectives and Major Themes” |
| Reviewer #1: Materials and institutional expertise: vague | We greatly thank the reviewer for this comment. We have included information specifying the affiliation of each member of the CoP in the text of the manuscript and included Table 1 to specify the materials, expertise, objectives, and goals used for the curricular thread.  “In fall 2022, faculty members and students of the DUSOM met to identify opportunities to integrate climate change and its health impacts within the UME. The meeting resulted in the creation of an interdisciplinary CoP of medical administrators, educators, students; faculty from the Nicholas School of the Environment, Schools of Nursing and Medicine, Center for Bioethics, Humanities, and History of Medicine, and Medical Center Library; community educators from the Museum of Life and Science, the Nasher Museum of Art, and Moms Clean Air Force. Importantly, the CoP’s medical educators included course directors of the first-year curriculum and medical students of all years, including student-leaders of the DUSOM Sustainability Committee and Environmental Justice and Health Equity Student Group. The CoP was tasked to develop and pilot a curricular thread, named Climate Change, Healthy and Equity, in the first-year Doctor of Medicine (MD) program by spring 2023.” | Page 6, lines 5-15  Table: page 19-21 |
| Reviewer #1: Assessment tools: Unclear how these were developed, given that no intervention has been developed, according to this description | According to Wald et al, critical reflection is a general tool used in curricular threads to evaluate students and provide individualized feedback. Ultimately, the goal of our curricular thread was to empower students consider acting on issues of climate change, health, and equity), and the first step of that is learning. According to Bloom’s taxonomy (Bloom et al. 1956, Adams et al. 2015), assessment of one’s own knowledge and synthesis and application of this knowledge is foundational to being able to have an impact. Our goal with the first year of the thread was to ensure that students could effectively synthesize their knowledge to become motivated to act, which is demonstrated by quotes from the essays and included in Table 1. As we intend that this thread will continue for many years, we will continue to build in more resources and opportunities for action and intervention.  We have added an Assessment sub-section under Methods, which includes information on the REFLECT rubric and the thematic analysis by Bloom’s taxonomy:  “Themes were organized according to Bloom’s Taxonomy into cognitive, affective, and psychomotor domains and further classified according to the socio-ecological model for health promotion into individual, institutional/relationship, societal, and policy factors.33, 34” | Line 2-5,  Page 10 |
| Reviewer #1: Where does the Health and Equity come from? This hasn’t been part of the intro. Is this what was meant as part of the ‘education need and expert recommendation?” | We thank the reviewer for highlighting this important point. One of the goals of our curricular thread was to emphasize equity. We have therefore added context and further detail to several areas of our manuscript to highlight the importance of understanding the connection between health equity and climate change and how we incorporated this into our thread.  Introduction:  “With the inevitable health consequences arising from climate change, medical students will interact with affected patients during their training and careers. Students are increasingly aware that the very healthcare systems they are members of contribute significantly to greenhouse gas emissions underlying anthropogenic climate change.1, 2 Moreover, while all individuals may be affected, communities that are historically marginalized, medically underserved, and older are at higher risk of experiencing the health impacts of climate change due to structural inequities.3, 4”  Methods:  “The main goals of Climate Change, Health, and Equity became to have medical students (1) understand the basic concepts of planetary health framework27; (2) identify effects of climate change on physical and mental health; (3) describe climate change as a social driver of health in the local community; (4) recognize examples of how climate policy disproportionately affects the health and equity of communities of color and of lower socioeconomic status; (5) recognize the importance of and learn from the lived experiences of communities disproportionately affected by climate change”  Table 1, Under Lecture 5:  “-Recognize the impact of climate change on health and health equity, specifically communities of color and lower socioeconomic status” | Line 5-11. Page 4  Line 4-10, Page 7  Table 1, Page 19 |
| Reviewer #1:  Also, it is hard to understand how the group, given the authors’ recognition of all the constraints preventing climate education, managed to get this thread into the curriculum. What seems to be needed here is more focus. Instead of starting the paper with all the problems, why not say something like (in a nutshell): “Although many institutions have confronted difficulties in incorporating climate change awareness into their busy curriculum, our medical school’s first-year climate, health, and equity course successfully addressed this barrier by integrating multiple urgent concerns students are confronting today. These include environmental threats, health equity, and [whatever other urgency the thread contains].” | We appreciate the reviewer’s comment. As we mention in our manuscript, we acknowledge that there has been development of ideas but challenge in action. Our interdisciplinary CoP was uniquely positioned as well as large enough to develop and integrate this thread because of prior similar threads existing in our curriculum and because of the varying expertise that CoP members had. We hope that outlining the affiliations and contributions of CoP members will make it possible for other institutions to adapt our curricular thread at their institutions.  “In fall 2022, faculty members and trainees met to address the lack of UME at our institution, Duke University School of Medicine (DUSOM), focused on climate change and its impacts on health. Subsequently, we created a community of practice (CoP) of medical educators and students. The CoP’s purpose was to identify areas within the first-year medical curriculum where topics of climate change could be integrated and to then develop a curricular thread in the first-year medical program with clear objectives and measurable outcomes. The resulting curricular thread was named Climate Change, Health, and Equity.”  “In fall 2022, faculty members and students of the DUSOM met to identify opportunities to integrate climate change and its health impacts within the UME. The meeting resulted in the creation of an interdisciplinary CoP of medical administrators, educators, students; faculty from the Nicholas School of the Environment, Schools of Nursing and Medicine, Center for Bioethics, Humanities, and History of Medicine, and Medical Center Library; community educators from the Museum of Life and Science, the Nasher Museum of Art, and Moms Clean Air Force. Importantly, the CoP’s medical educators included course directors of the first-year curriculum and medical students of all years, including student-leaders of the DUSOM Sustainability Committee and Environmental Justice and Health Equity Student Group. The CoP was tasked to develop and pilot a curricular thread, named Climate Change, Healthy and Equity, in the first-year Doctor of Medicine (MD) program by spring 2023.” | Line 13-19.  Page 5  Line 5-15, Page 6 |
| Reviewer #1:  p. 3 evolves from? You must mean ‘evolves beyond’ or something like that | We agree with the reviewer that evolves was not the appropriate word to use. We have revised this sentence to be clearer and use the word “overcomes” instead. | Page 5, line 22 |
| Reviewer #1: “We intended…humanities” The reader recognizes this as a big ask and hopes that what follows will illuminate how these goals were addressed | We appreciate this comment and have revised this sentence to be more targeted to what we proposed and accomplished in this curricular thread.  “In addition to foundational lectures, we intended to foster the growth of medical students’ critical consciousness using facilitated discussions, self-reflection and writing exercises, and experiential learning activities focused on informed advocacy, personal accountability, and environmental health justice.” | Line 20-23, Line 1-3,  Page 5-6 |
| Reviewer #1: p. 4. The power of the course directors is key here; the lack thereof would be a major obstacle at other institutions | We agree with the reviewer that the course directors, who were study authors, were important for the thread development and integration. We feel that any curricular innovation would require the guidance of course directors. |  |
| Reviewer #1: “118 students engaged in thread content”: isn’t there a better way to say this? How about: “The course thread included…” you can mention the 118 students later, probably not necessary until the results | Thank you for this comment. We have moved the description of the number of participants to the results section. It now reads:  “One hundred percent of the 118 first-year medical students who participated in the thread submitted an essay, and none opted out of inclusion in the analysis.” | Line 15-16,  Page 10 |
| Reviewer #1: “All thread content was developed” It seems as though you are going backwards at best or all over the place at worst. Reorder to state: Who developed thread content, how it was integrated (as lectures etc); and additional content (2 mandatory sessions; 2 community engagement sessions). | We thank the reviewer for this insightful comment, and we agree with the reviewer that the thread development is best described in chronological order for the sake of clarity. For this reason, we have edited the section subheadings and content to reflect 1) the needs assessment and creation of the community of practice, 2) the thread development, and then 3) assessment. | Pages 7-10 |
| Reviewer #1: Interactive and community sessions: It is unclear how these matter to the focus of your paper. You say too little about their relevance. I would just mention them, maybe with the briefest possible mention in the context of the overall climate and equity intention of the course. All you need to say without getting into deep water is “Exhibits addressing the [physical and emotional?] impacts of climate change on affected communities were well-suited to provoking thoughtful discussions.” | We thank you for this comment. We have updated the text descriptions to briefly describe the content of the exhibits that students were guided through as part of the experiential learning activities. The section reads:  “For the two experiential learning activities in the community, members of the CoP organized guided tours and presentations at the Museum of Life and Science and Nasher Museum of Art. Students were guided through special exhibits on the climate crisis. The goal of the tour was to stimulate perspective-taking by considering the diverse lived experiences within local, national, and global communities, and imagining the impact of climate change on the health of subjects within art pieces and installations. Opportunities for extracurricular involvement were advertised in-class and by email, and included symposiums hosted by national and local organizations and volunteer events with the Environmental Justice and Health Equity Student Group.”  Additionally, descriptions of the experiential learning activities as well as their objectives and themes have been added to Table 1 (“Overview of curricular thread activities”). | Lines 12-20, Page 8  Table: 19-21 |
| Reviewer #1: Overall: consider what the reader needs to understand about the basic course structure and intent. In terms of all the details, the reader won’t have the same local resources you have; if they think they might be able to apply your approach to their own resources, they can contact you. | Thank you for this comment, as we believe it is critical that these efforts can be replicated at other institutions. We have included more detail about each lecture and activity in the thread in Table 1 (“Overview of curricular thread activities”). We hope that these more detailed descriptions of thread lecturers and content will allow other universities to find experts and exhibits to address similar themes in their local area. | Table: 19-21 |
| Reviewer #1: It’s unclear how beliefs about climate change are a ‘metareflective exercise of their learning.” The reader has no idea from this sentence what your goal is. Also if they were required to write this, how come they had the option to opt out? It sounds contradictory. Maybe: any student who did not choose to opt out was required… | We agree with the reviewer that this was not a clear statement. Rather than using the term “metareflective”, we have changed this section to state that:  “At the conclusion of the thread, students were given two prompts designed by the CoP and intended to probe changes in preexisting beliefs and attitudes, motivation to continue learning about climate change as a social driver of health, and likelihood of participating in advocacy and community engagement.”  Regarding the reviewer’s comments on why students had the option to opt out: all students were all required to complete the essay as part of FP2C course. However, they could opt out of having their essay used for thematic analysis in this research. This has been clarified in the “Study Approval” section. | Line 22-23,  Page 8  Line 5-12,  Page 10 |
| Reviewer #1:  p. 7 There seems to be an overlap between methods and results. Methods should state what the overall plan and process is. The results should state how many people participated, if any did not participate, and what was found. | We thank the reviewer immensely for this comment and have made substantial revisions to our methods and results section to be succinct and clearer.  We have revised the methods to have four subsections: 1) Needs Assessment and Creation of the Community of Practice, 2) Thread Development, 3) Assessment, 4) Study Approval.  We have also moved all statements about participation into the Results, beginning with the first sentences: “One hundred percent of the 118 first-year medical students who participated in the thread submitted an essay, and none opted out of inclusion in the analysis. Thirty of the 118 submitted essays (25% of the total sample) were randomly chosen for assessment by the REFLECT rubric and for the thematic analysis by Bloom’s Taxonomy...” | Page 6-10  Page 10, lines 15-18 |
| Reviewer #1:  Nothing is said about the thematic analysis itself and how it was conducted. See: Kiger ME, Varpio L. Thematic analysis of qualitative data: AMEE Guide No. 131. Med Teach. 2020;42(8):846-854. | We apologize for any lack of clarity on the assessment of student essays. We have therefore added information under the Assessment sub-section in the Methods section, which reads:  “We used a sample of 30 essays, approximately 25% of the total submissions, with the expectation that this sample size would be sufficient to reach saturation, or identification of most unique themes.30-32 Submitted essays were deidentified and assigned a number. A random number generator identified 30 numbers, and the corresponding essays were used in the analysis. Study authors TD and EA graded the selected essays according to the REFLECT rubric. As a standard setting practice, TD and EA reviewed four essays blindly and then together. The remaining essays were reviewed and graded independently. Similarly, study authors SM and PM, who are trained in narrative medicine, performed the thematic analysis of the selected essays. Study author SM is a faculty member at DUSOM but not directly involved in this thread, and PM is a faculty member at an external United States health sciences university. SM and PM reviewed five essays together to develop a codebook, and the remaining were reviewed and coded independently. Themes were organized according to Bloom’s Taxonomy into cognitive, affective, and psychomotor domains and further classified according to the socio-ecological model for health promotion into individual, institutional/relationship, societal, and policy factors.33, 34” | Line 14-23,  Line 1-5, Page 9-10 |
| Reviewer #1:  7-8 “For instance, one student connected a lecture statistic regarding the nature of poor air quality ..with environmental pathology…” The reader is unlikely to understand what the actual insight here is, so I suggest finding a better, less obscure-sounding example | We agree that this quote, although impactful as written in the essay, was not given enough context. We have removed this quote from the text and chose instead to highlight common themes touched upon by students in each of the three Bloom’s taxonomy domains. Specific quotes reflecting each of these themes have been added to Tables 2-4. | Page 22-27 |
| Reviewer #1:  “…expand their medical knowledge…to include a critical review of the literature” It’s unclear how the above examples, which are pretty basic, show this. Wouldn’t students have heard in their sessions about the disparities mentioned in the quoted material? | The authors agree that it was unclear what information students learned in the course content versus in their own independent literature review. Students received a lecture from a medical librarian that helped them to explore primary literature on health and climate change. Students were required to use these skills to find a primary source that connected health and climate change as part of their self-directed learning activity. For clarity, this required component of the self-directed learning exercise has been added to the Methods section under thread development. It now reads:  “The lecture on critical analysis of primary literature was led by study author SK, a Research and Education Liaison Librarian at DUSOM. The objective of the session was to teach students how to effectively search interdisciplinary and subject-specific databases relevant to climate change research to identify literature on climate change its health impacts and locate clinical decision-making tools. Following completion of the session, students were tasked with completing a self-directed learning activity in which they independently accessed, selected, and critically analyzed a primary literature source examining a lived experience related to climate. This activity aligned with LCME standard 6.3 for Self-Directed and Lifelong Learning,28 and the findings will be reported in a separate paper.” | Line 3-11, Page 8 |
| Reviewer #1:  9 ”…importance of hearing directly from affected communities” It’s unclear whether students were asking for such contacts, or whether in the course they had such contacts. Or were they responding to the museum exhibit? | Students learned about the impact of climate change on underserved communities in both lecture content and the experiential learning activities. In the essays, most students referred to the museum exhibit, so this sentence has been clarified to:  “From the museum exhibits, students reflected on the affective importance of including perspectives that are often excluded from conversations centered around the environment, including Indigenous peoples, urban residents, and rural farmworkers.”  We have also added direct quotes to contextualize this theme in Table 2 and 3:  Under Table 2 “Identifying effects of climate change on equity”  Under Table 3 “Recognizing the impact climate change has on societal inequities” | Line 22-23. Line 1-2  Page 11-12  Page 22-25 |
| Reviewer #1:  Psychomotor: It’s unclear how much content in the teaching thread addressed actionable items | We agree with the reviewer that it was not clear what domains each lecture and activity addressed. Therefore, we have included a column in Table 1 that identifies which domains were addressed. Additionally, we have included quotes from the student essays that involve the psychomotor domain. | Page 19-21  Page 26-27 |
| Reviewer #1:  10 “Students participated in foundational lectures…on attitudes and beliefs” I feel that this should be embedded in the method section or tabulated in the results section. This is not the place to mention this as though it were some sort of concluding insight | The authors agree that the description of how students’ essays were evaluated for “reflection” was unclear. We have updated the description of the methods used to determine whether students were “critically reflecting” in their essays, which was evaluated using the REFLECT rubric. The results of this analysis are now shown in Figure 1 and discussed in “Results”. | Line 14-18,  Page 9  Line 18-20, Page 10 |
| Reviewer #1:  “resulted in calls to action” There is a big difference between calls to action as expressed in an assigned essay vs in an activist format. “Students reflected in their essays on the different ways students could actively engage” seems more accurate. Better yet: “These activities ultimately led students to express a need for action and a desire to learn from the lived experience of patients affected by climate change. Through critical reflection…”  However, I don’t think you can claim that students generated a sense of personal & professional responsibility…etc. The more accurate claim would be that they expressed this sense. | We agree with the reviewer’s comment and therefore have revised the language of this statement to:  “Nearly every essay (24/30; 80%) included statements expressing the need for action (Table 4) at the individual level … and/or the institutional level… Lastly, students acknowledged the power of collective influence and the sense of responsibility they felt following participation in the curricular thread.” | Line 5-16,  Page 12 |
| Reviewer #1:  It’s unclear to me how the CoP transformed the topic of climate change. Nothing you describe really supports this. And for whom do you mean? For the first year students or the CoP members? If the latter, you don’t show it in the paper. If the former, all we have is the claims in their essays. Maybe the students did express something like “This course really transformed my thinking” but you don’t provide evidence of that. If you mean the CoP members, we really don’t hear anything in this paper about the impact on them. | We appreciate this comment which highlights the lack of clarity in our writing. The CoP’s goal was to transform the topic of climate change for medical students. To highlight this directly, we have included quotes from student essays.  Examples in the Tables now include:  “Before the climate change thread, I also largely thought of climate change in terms of natural disasters or dramatic weather events; however, it is critical to consider the daily impact of climate change. Thus, **the climate change thread helped me expand my view** to consider the daily impact of climate change on rural populations that we will be serving during our clerkship year.”  “This **thread has been incredibly insightful** as to how climate change impacts our collective health while also disproportionately disadvantaging those from lower-income areas and historically socio-politically underrepresented communities.”  “**Prior to this thread**, I had an understanding that climate change had drastic effects on population health and wellbeing. However**, I don’t think I fully understood how it is a social determinant of health**.” | Table 2-3, Page 22-25 |
| Reviewer #1:  “Holistic educational approach towards addressing inequities…” This doesn’t seem to have been the goal of this paper as stated in the intro | Thank you for this comment. This sentence has been removed from the discussion. |  |
| Reviewer #1:  “Didactics were augmented…etc” Why is this in the discussion? This seems like it should be in methods or not directly relevant to the paper. | Thank you for this comment. The authors intended to emphasize that the thread included experiential learning activities in the community that would allow students to connect the themes in the didactic lectures to community health. This was intended to enable students to seek opportunities for advocacy related to climate justice in the community. For clarity, we have specified these goals in the text.  “Through dynamic conversations centered on the current and future impacts of climate change on health, we created foundational lectures, guided discussions, organized experiences in the community, and facilitated self-reflection on learner’s attitudes and beliefs. This multi-pronged approach ultimately led students to express both the need for action and their desire to further learn from the lived experiences of patients affected by climate change in their self-reflection essays. Uniquely, we included self-reflective and experiential learning activities after students in the CoP voiced that seeing tangible effects of climate change on the health of communities may invoke desires to continue learning and advocating beyond the curricular thread.” | Line 21-23, Line 1-6,  Page 12-13 |
| Reviewer #1:  p. 11 What are ‘unanimous reflections?” Do you mean, nearly all participants highlighted the impact the thread had in furthering reflection on the roles of privilege, responsibility and action? | Thank you for the comment, and we agree that this phrase required clarification. We have added counts of how many of the essays addressed each of the Bloom’s taxonomy domains, and these numbers indicate that nearly all students touched on the affective and psychomotor domains. We highlight responsibility, and action as commonly mentioned themes within those domains. We did not quantify the prevalence of these themes, but we have added quotes from student essays addressing these and other themes in Tables 2-4. | Line 1,  Page 11  Line 15,  Page 11  Line 5,  Page 12  Table 2-4, Page 22-27 |
| Reviewer #1:  Future Directions: Condense the first three sentences to ‘long-term impact’ on knowledge and action, as these plans seem unformed/speculative as yet | Thank you for this comment. These sentences have been condensed and now read:  “For future directions, we plan to measure the long-term impact of the thread on knowledge and action.” | Line 7-8, Page 14 |
| Reviewer #1:  Adapting experiential community-based activities: I don’t know if the readers really understand what the authors did in their museum visits. The alternative suggestions are potentially great, but it’s not that easy for educators to adapt them, especially without knowing the details of what the specifics of such community exhibits are—not to mention in what context they could make demands of this sort on the curriculum. Maybe a Table detailing examples and specific, itemized suggestions would help | The authors agree that specific themes of these activities are necessary for other institutions to replicate these activities locally. We have therefore added the following description to our Methods:  “For the two experiential learning activities in the community, members of the CoP organized guided tours and presentations at the Museum of Life and Science and Nasher Museum of Art. Students were guided through special exhibits on the climate crisis. The goal of the tour was to stimulate perspective-taking by considering the diverse lived experiences within local, national, and global communities, and imagining the impact of climate change on the health of subjects within art pieces and installations.”  Descriptions of the experiential learning activities as well as their objectives and themes have been added to Table 1 (“Overview of curricular thread activities”). We hope that these more detailed descriptions of thread lecturers and content will allow other universities to find experts and exhibits to address similar themes in their local area. | Lines 12-17,  Page 8  Table 1, Page 19-21 |
| Reviewer #2:  Minor grammatical suggestions: Add a comma in the first line of the abstract.  …impact on the environment and health, medical schools...  Page 1: Education on climate change’s affect (change to effect) on health... | We have revised the manuscript for grammatical errors and have changed “affect” to “effect”. | Page 4, line 22 |
| Reviewer #2:  Methods: How and why are 30 essays chosen for thematic analysis? Is 30 a representative number of essays here? Would suggest to add more information regarding this in the methods section. | We thank the reviewer for this important question. We admit that choosing a sample size for qualitative research is a challenging, and as Vasileiou et al. states, often without justification. We chose 30 essays as this was 25% of the total number of essays submitted. Studies on qualitative research have found that anywhere from 9-17 interviews (Hennik et al. 2022) or 16-24 interviews (Hennik et al. 2016) are needed for “saturation”, where consistent thematic elements have been identified without a need to screen additional interviews. We have added the following detail to the paper to make clear to readers of the reasoning behind this choice:  “We used a sample of 30 essays, approximately 25% of the total submissions, with the expectation that this sample size would be sufficient to reach saturation, or identification of most unique themes.30-32” | Line 14-16, Page 9 |
| Reviewer #3:  General question -- The invitation from PLOS ONE reads as follows:  I am inviting you to peer review the following manuscript for PLOS ONE. In view of your expertise, I would greatly value your input on this manuscript’s suitability for publication.  Manuscript #: PONE-D-23-31947  Title: Fostering medical students’ critical consciousness on climate change, health, and equity  Authors: Andrew Muzyk; Trisha Dalapati; Sneha Mantri; Phillip Mitchell; Ian Allen George; Kathryn Andolsek; John Matthew Velkey; Jennifer Lawson  Article type: Research Article  So, why are you anonymizing the manuscript, e.g., Study authors XX and XX were responsible for the creation and implementation of the Climate Change, Health, and Equity thread in the first-year medical school pre-clerkship curriculum at the XXSOM and elsewhere. Makes the manuscript harder to read. | We thank the reviewer for bringing this discrepancy to our attention. We had anonymized the manuscript without realizing that the identify and affiliations of the authors would be made available during the peer review process. We have revised the manuscript accordingly (ie. XX School of Medicine (XXSOM) to Duke University School of Medicine (DUSOM). |  |
| Reviewer #2:  2. Table 1 provides a summary but no details regarding the cognitive, affective, and psychomotor domains and further classified according to the socio-ecological model for health promotion into individual, institutional/relationship, societal, and policy factors.  A. The manuscript itself should include quantitative data regarding frequencies.  B. Either the manuscript or an appendix should include actual examples that were interpreted according to the different categories. | Thank you for this comment, and we agree that further details regarding how essays addressed Bloom’s taxonomy domains improves the manuscript. We have split the themes addressed in many students’ essays into individual, institutional/relationship, societal, and policy categories. We have provided examples of student quotes for each theme in Tables 2-4.  We have also added a count of how many essays addressed each of the three domains in the “Results” section. | Table 2-4, Page 22-27  Line 1,  Page 11  Line 15,  Page 11  Line 5,  Page 12 |
| Reviewer #2:  3. What is the control? Are the students’ answers a consequence of the Climate Change, Health, and Equity thread in the first-year medical school pre-clerkship curriculum or would 2nd year or more advanced students who didn’t participate in the thread have provided similar answers? | We agree with the sentiments of the reviewer regarding the importance of including a control group. The curriculum at Duke University School of Medicine includes only one year of didactic work so it was not feasible to include reflections from second year students, who are on clinical rotations and who did not participate in the thread. The lack of a control group is a limitation of our research study, and we have added information to acknowledge this:  “Longitudinal follow-up is especially critical as our school’s curriculum consists of only a single pre-clerkship year. Our cohort was limited to these first-year students, and we did not have a control group of students to compare reflection outcomes to.”  We hope that the quotes from the student essays in Tables 2-4 highlight that many participants felt changes in their cognitive, affective, and psychomotor domains as a direct result of the thread. | Line 8-10,  Page 14  Page 22-27 |
